# Behavioral and Neural Correlates of Executive Function: Interplay between Inhibition and Updating Processes

**DOI:** 10.3389/fnins.2017.00378

**Published:** 2017-06-30

**Authors:** Na Young Kim, Ellen Wittenberg, Chang S. Nam

**Affiliations:** Edward P. Fitts Department of Industrial and Systems Engineering, North Carolina State UniversityRaleigh, NC, United States

**Keywords:** working memory, executive function, EEG, granger causality, effective connectivity, cognitive control network

## Abstract

This study investigated the interaction between two executive function processes, inhibition and updating, through analyses of behavioral, neurophysiological, and effective connectivity metrics. Although, many studies have focused on behavioral effects of executive function processes individually, few studies have examined the dynamic causal interactions between these two functions. A total of twenty participants from a local university performed a dual task combing flanker and n-back experimental paradigms, and completed the Operation Span Task designed to measure working memory capacity. We found that both behavioral (accuracy and reaction time) and neurophysiological (P300 amplitude and alpha band power) metrics on the inhibition task (i.e., flanker task) were influenced by the updating load (n-back level) and modulated by working memory capacity. Using independent component analysis, source localization (DIPFIT), and Granger Causality analysis of the EEG time-series data, the present study demonstrated that manipulation of cognitive demand in a dual executive function task influenced the causal neural network. We compared connectivity across three updating loads (n-back levels) and found that experimental manipulation of working memory load enhanced causal connectivity of a large-scale neurocognitive network. This network contains the prefrontal and parietal cortices, which are associated with inhibition and updating executive function processes. This study has potential applications in human performance modeling and assessment of mental workload, such as the design of training materials and interfaces for those performing complex multitasking under stress.

## Introduction

Most daily tasks require the ability to mentally process and maintain information, especially when one is trying to adapting to activities where practiced cognitive processes are not useful (Nee et al., 2013). Some examples include planning for a road trip, performing mental math, playing games such as chess, and completing a project on time and under budget. Each of these tasks requires flexible, long-term thinking, recalling knowledge, and inhibiting distractions, which are collectively termed executive function (EF). EF is defined as “processes necessary to control or regulate other cognitive processes in the service of goal-directed behavior” (Minzenberg and Laird, 2009, p. 3). Since EF is defined as a group of processes, there has been a focus on fractionating this concept into three distinguishable cognitive processes: (1) *updating*, coding incoming information for task relevance, then revising the items in working memory by replacing no longer relevant information with more relevant information (Morris and Jones, 1990), (2) *shifting*, switching between multiple tasks, operations or mental sets (Monsell, 1996) and (3) *inhibiting*, controlling the suppression of prepotent responses (Baddeley, 1996a,b; Miyake et al., 2000). However, fractionation of executive function led to further investigation into whether these components shared a common, underlying mechanism (unity hypothesis), or if they were completely separable (diversity hypothesis) (Miyake et al., 2000; Jurado and Rosselli, 2007).

Behavioral studies investigating performance on various complex cognitive tasks (e.g., Wisconsin Card Sorting Task) or the correlation between performance on multiple EF tasks have shown that there are individual differences in EF ability, with subjects performing well on some complex tasks but not others or poor correlation between the single EF tasks (Lehto, 1996; Godefroy et al., 1999). Further investigation into these research paradigms revealed that these studies are limited by the impurity problem. That is, because EFs operate on other cognitive processes, it is difficult to design paradigms that directly target a single component (Miyake et al., 2000). Miyake et al. (2000) aimed to address the EF unity or diversity question. To avoid the impurity problem, simple cognitive tasks were identified so that each task used only one primary EF (see Table 1). Behavioral performance measures on these tasks for 137 college students were analyzed via latent variable analysis, leading to the conclusion that there is evidence that updating, shifting, and inhibiting share a common underlying process. It has been suggested that this common underlying process is controlled attention (Engle, 2002). Furthermore, neuroimaging studies using similar simple EF tasks, as listed in Table 1, indicated that EF components share a common brain network, further supporting the unity hypothesis (Niendam et al., 2012).

**Table 1:** Summary of simple cognitive tasks used to investigate individual executive functions.

**Executive function**	**Tasks from Miyake et al. (2000)**	**Other tasks (Niendam et al., 2012)**
Shifting	• Plus-Minus	• Sorting Tasks
	• Number-Letter	
	• Local-Global	
Updating	• Keep Track	• N-back
	• Tone Monitoring	• Sequence Recall
	• Letter Memory	• Sternberg Task
Inhibition	• Antisaccade	• Erikson Flanker Task
	• Stop-Signal	• Simon Task
	• Stroop	• Go/No-Go Task

Given that both behavioral and neuroimaging evidence supports the unity of the EF components, there are additional questions regarding the interaction between the three EF components (shifting, updating, and inhibition) and the underlying mechanism (i.e., controlled attention) unifying these three components.

There are two opposite hypotheses which would show two possible results: (1) the use of multiple EFs, requiring increased attentional control, will enhance performance or (2) it will diminish performance (de Fockert, 2013). Determining which of these two hypotheses is supported has important implications in human performance modeling, that is, do tasks requiring intricate shifting, updating and inhibition to achieve task goals result in better performance than simpler tasks requiring less executive function, such as following simple instructions in a distraction-free environment? The present study focused on the relationship between EFs, specifically aiming to investigate the implications of modulating updating load on the inhibition process. Simple cognitive tasks (i.e., n-back and Erikson Flanker tasks) were employed to appropriately and independently manipulate both updating and inhibiting EF components.

Important insights into the relationships between task difficulty (the use of multiple EF, increased attentional load) and performance is provided by dual-task (DT) paradigms that modulate working memory (WM) load on single EF tasks (Vandierendonck, 2014; Gil-Gómez de Liaño et al., 2016). DT studies investigating the impact of WM load on the inhibition EF component utilize the Eriksen Flanker task (Eriksen and Eriksen, 1974), where the subject is required to distinguish between incongruent trials, in which the middle, target stimulus conflicts with the flanking, distractor stimuli (e.g., < < > < <) and congruent stimuli (e.g., < < < < <), and Stroop task (Stroop, 1935), where the subject must inhibit meaning of the word and respond with the color of the text. Using the flanker task, Lavie et al. (2004) found support for diminished performance under high loads. In the DT condition, a varying number of probes were presented for future recall after completion of the flanker task, which resulted in a performance decrement when compared to single task performance, suggesting that it is more difficult to inhibit the incongruent flanker stimuli when WM is loaded. This was interpreted as a depletion of common attentional control processes necessary in both tasks, resulting in poor suppression of irrelevant flanker stimuli in the DT condition (Lavie et al., 2004). While this study provided insight on the impact of WM load on inhibition, it did not include a quantitative measure of attentional resources. Thus, it is still unclear how the authors can conclude that attentional resources were depleted for all subjects in the DT condition.

Conversely, in Kim et al. (2005) performance under high loading was shown to enhance performance on the Stroop task. In the condition where WM load and the distractor (an additional stimulus) were in the same modality, Stroop performance was improved. The authors interpreted these results as an increase in activation of attentional processes under high WM load, which serve to facilitate inhibition. Again, this study investigated impact of WM load on inhibition but did not include quantitative measures of WM capacity. As can be seen in these inhibition tasks, there was no consensus on the impact of WM load on EF performance; there are studies that report that an increase in WM load leads to reduced performance (e.g., Lavie et al., 2004), little to no impact on performance (e.g., Woodman and Luck, 2007), and improved performance (e.g., Kim et al., 2005). The updating EF is commonly evaluated using the n-back task, where the subject is required to respond to the target stimulus only if it matches the stimulus presented n-levels previously. Updating demand is modulated by increasing the n-back level; higher n-back levels increase task difficulty as compared to lower n-back levels (Owen et al., 2005).

For both inhibition and updating tasks, reaction time and accuracy are indicators of behavioral performance. In single task conditions, increased updating demands in high n-back levels result in increased RTs and decreased accuracy as compared to low n-back levels (Jaeggi et al., 2010). Likewise, incongruent Flanker trials results in increased RTs and decreased accuracy compared to congruent Flanker trials because they require increased inhibitory control (Eriksen and Eriksen, 1974). Additionally, inhibition and updating tasks have been observed to impact neurophysiological measures, primarily the amplitude of the event related potential (ERP) around 300 ms post-stimulus presentation (P300), an indicator of internal attention distribution, and the alpha band power (Watter et al., 2001). Gevins and Smith (2000) observed that theta power increased and upper alpha power decreased under higher cognitive workloads. Additionally, Scharinger et al. (2015) demonstrated that upper alpha band power was modulated by cognitive workload level (n-back level). However, this group did not observe a significant difference in theta power with change in n-back level. Therefore, this study is limited to analysis of mean upper alpha band power. In n-back paradigms, alpha band power decreases at parietal electrodes and P300 amplitude decreases at increased n-back levels (Gevins et al., 1997). In incongruent flanker trials, P300 amplitude decreases compared to congruent trials (Pratt et al., 2011). Also, trials that require increased attentional control, such as in the incongruent flanker trials, haven been shown to decrease alpha band activity in the parietal lobe (Alfonso et al., 2013).

Lastly, studies that couple EF tasks with high spatial resolution neuroimaging methods, such as functional MRI, have identified areas of the brain responsible for attentional control and EF. In a recent meta-analysis of 193 neuroimaging studies, Niendam et al. (2012) identified activation of the cognitive control network (CCN) in the frontal and parietal areas for both inhibition and updating tasks. This network includes the dorsolateral prefrontal cortex (Brodmann Area or BA 9, 46), anterior cingulate cortex (BA 32), superior and inferior parietal lobe (BA 7, 40), prefrontal cortex (BA 6, 10), temporal cortex (BA 13), occipital cortex (BA 19). Also, activation was seen in the subcortical regions including the thalamus, caudate, putamen, and cerebellar declive. The meta-analysis also identified activation of the cingulate (BA 32, 24) and temporal (BA 13, 37) cortex unique to WM and updating tasks (Niendam et al., 2012). Scharinger et al. (2015) combined n-back and flanker task stimuli into a single-task paradigm to investigate the relationship between inhibition and updating EFs. This study found that flanker task performance was improved in trials with increased updating load for both behavioral and neurophysiological measures, which supports the findings of Kim et al. (2005). Scharinger et al. (2015) interpreted their results as increased activation of the attentional network under increased updating load, leading to enhancement of inhibitory control and improved performance. However, their analysis was limited to correlation of brain activity (i.e., P300 and alpha power) between conditions and did not explicitly address the mechanism underlying the EF relationship. That is, they did not localize the sources of brain activity or investigate the directional relationships that would support an increase in activation.

The goal of this study was to investigate the relationship between inhibition and updating EF processes at behavioral, neurophysiological, and neural connectivity levels. A dual-task paradigm combining flanker and n-back stimuli was used to modulate updating load and evaluate inhibitory control. All subjects were categorized by the working memory capacity, as measured by the operational span task (OSPAN), to address the shortcomings of previous studies with regard to the depletion/availability of attentional resources (Turner and Engle, 1989). Additionally, this study expanded on Scharinger et al. (2015) by exploring brain activity beyond correlation relationships by measuring effective connectivity. Specifically, Granger Causality analysis (GC) was used to determine the directed causal dependencies of brain regions from the EEG signal. GC is a method for investigating whether one time-series correctly predicts another and allows us to analyze brain circuit connections and how they change over the course of a cognitive process (Coben and Mohammad-Rezazadeh, 2015). We hypothesized that, similar to Scharinger et al. (2015), an increase in updating load would affect both behavioral and neurophysiological measures and these metrics would be modulated by individual working memory capacity (i.e., OSPAN score). Additionally, we hypothesized that source localization would identify neural sources within the CCN in the frontal and parietal areas. Lastly, we hypothesized that the effective connectivity between high updating load conditions would exhibit enhance activation of the CCN as compared to the connectivity observed in low load conditions.

## Methods

### Participants

A total of twenty participants (8 male; 12 female) from a local university participated in the present study. Participants were given monetary compensation for their participation. All participants successfully completed the entire experiment and were included in the data analyses, whose mean age was 21.8 years (standard deviation, *SD* = 2.67). Participants reported being free of any medical or neurological disorders and had normal or corrected vision. Participants gave their written consent after a detailed explanation of the experiment procedure which was reviewed and approved by the University's Institutional Review Board.

### Stimuli and experimental task

**Figure 1:**
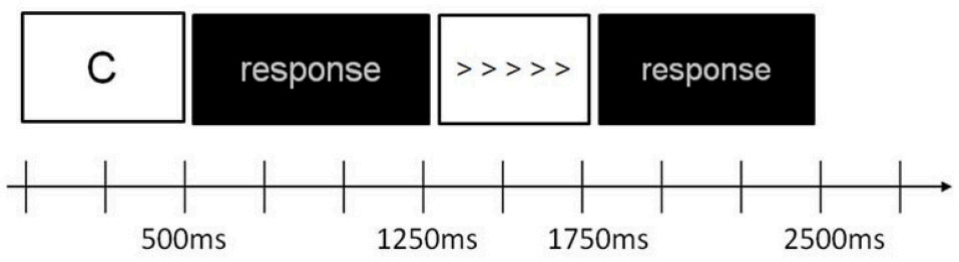
Schematic of stimulus sequence and timing of the n-back task with flanker stimuli for 1 trial.

An arrowhead version of the flanker task (Eriksen and Eriksen, 1974; Kopp et al., 1996) was combined with a working memory n-back task (Braver et al., 1997). Seven letters were used as stimuli in the n-back task (C, G, H, K, T, Q, or W) (Klawohn et al., 2016). In other words, two types of stimuli (n-back and flanker) were interleaved on each trial. At the beginning of each trial, one letter was displayed for 500 ms, followed by a black screen for 750 ms, and then flanker stimuli was presented (five horizontal arrowheads) for 500 ms, followed by a black screen for 750 ms. The total duration of each trial, regardless of letter or flanker stimuli, was 1,250 ms. Refer to Figure 1 for stimulus presentation and timing for one trial.

There were three n-back conditions (0-back, 1-back, and 2-back). Six blocks, two at each n-back condition, were completed by each participant in a counterbalanced order. Each block consisted of one n-back level. Before the beginning of each block, the instructor let them know the difficulty level of n-back conditions. The stimuli sequence for each block had an equal number of n-back and flanker stimuli (120 trials each). Congruent and incongruent flanker stimuli were presented with equal likelihood for each block and n-back stimuli consisted of 20% target and 80% non-target letters (Klawohn et al., 2016). The participants were instructed to respond quickly and accurately to the appropriate n-back target stimuli. Furthermore, subjects were instructed to press either left or right buttons with their left or right index finger based on the direction of the mid position arrowhead, irrespective of the letter stimuli presented in between (Klawohn et al., 2016). Each block lasted ~5 min, and the entire testing procedure, including breaks, lasted ~50 min.

### Apparatus and materials

#### Operation span task (OSPAN)

One of the common working memory span tasks that is used to capture the cognitive construct of working memory is the Operation Span task (Turner and Engle, 1989; Liu et al., 2016). The OSPAN has proven to be a reliable and valid indictor of overall working memory capacity (Conway et al., 1999; Redick and Engle, 2006). The present study employed the automated version of the Operation Span task to assess participants' overall working memory capacity, because the automated OSPAN can quickly administered, completely computerized, and automatically scored (Unsworth et al., 2005). The automated OSPAN consisted of three practice sessions (letter span, math problem, both of them combined) and one experimental session with 75 trials. For each experimental trial, participants first see a math equation, then indicate whether the presented answer is correct, and finally remember a letter for later recall. The present study adopted an 85% accuracy criterion on the math operations for all participants in order to ensure that participants were not trading off between solving the math operations and remembering the letters. The entire task took ~20 min to complete (Unsworth et al., 2005).

### EEG acquisition and pre-processing

EEG signals were recorded using an EEG cap (Electro-Cap International, Inc.) embedded with 48 active electrodes covering frontal, central, parietal and occipital areas, based on the modified 10–20 system of the International Federation (Sharbrough et al., 1991). Recordings were referenced to the left ear lobe and grounded to between AFz and Fpz. EEG signals were amplified with a g.USBamp amplifier (g.tec Medical Engineering). EEG signals were sampled at 512 Hz and band-pass filtered between 0.01 and 75 Hz to take out unwanted frequency bands, and notch-filtered at 60 Hz to remove US electrical mains hum.

EEG data was epoched using event-locked time windows, ranging from 500 ms proceeding subject response (button press) to 1,250 ms following subject response. EEG data were visually inspected to exclude trials that contained electrode drift noise and muscle movement-related noise. Independent component analysis was used to decompose the EEG signal into independent components (ICs). All ICs were visually inspected and components that resembled EOG activity were rejected from further analysis. Signal acquisition and processing were all conducted using BCI2000 system (Schalk et al., 2004), MATLAB (The MathWorks), and EEGLAB (Delorme et al., 2011).

### Experimental design and independent variables

The experiment design in this study consisted of a 3 (updating demand level) x 2 (inhibition demand level) within-subjects design.

#### Updating demand level

The necessity of updating working memory was manipulated using an n-back task with three difficulty levels (0-, 1-, and 2-back). In the n-back task, participants are required to decide whether a currently presented stimulus matches the stimulus previously presented “n” trials back. In the 0-back condition, participants were instructed to respond to a single predetermined target stimulus (e.g., “G”). In the 1-back condition, the target was defined as a letter that was identical to the one immediately preceding it (e.g., one trial back). In the 2-back condition, the target was defined as a letter identical to the one which was presented two trials back. Participants pressed a button for targets (~20% of trials) with their dominant hand (Klawohn et al., 2016). The n-back paradigms have been employed in many human studies to investigate the neural basis of executive functions, in particular working memory, because the task requires on-line monitoring, updating, and manipulation of remembered information and is therefore assumed to place great demands on key processes within working memory (Owen et al., 2005).

#### Inhibition demand level

Two levels of the inhibition demand (congruent vs. incongruent) were manipulated using the flanker task, which is traditionally used to measure the efficiency of executive function processes due to conflicts in the stimuli presented (Eriksen and Eriksen, 1974). The flanker task requires participants to evaluate the direction of the center, target arrow and the flanking arrows, which present cognitive interference (Nigbur et al., 2011). Flanker stimuli were either congruent (i.e., target and flanker arrows point in the same direction) or were incongruent (i.e., target and flanker arrows point in opposite directions (Kopp et al., 1996). Additionally, participants were asked to press a button only for incongruent flanker trials, thus presenting response conflict. Inhibition was required to successfully overcome the cognitive interference between the target and flanker stimuli and accurately selected the appropriate response.

### Dependent variables and data processing

To investigate the roles of two EF processes (updating and inhibition), this study measured several data at behavioral, neurophysiological and neural connectivity levels.

### Behavioral data

Signal detection accuracy and reaction times were evaluated for both the flanker and n-back tasks at each n-back level.

#### Accuracy (%)

In the flanker tasks, a hit was a correct response and a false alarm (FA) was an incorrect response or an omission of a response to a stimuli. Flanker task hit, FA, correct rejection, and miss rates for each n-back level were averaged over all participants. In the n-back tasks, a hit was a response to the appropriate target, while a FA was a response to an incorrect target. Hit, FA, correct rejection, and miss rates for the n-back task were averaged over all participants at each n-back level.

#### Reaction time (RT, millisecond)

For each subject response, reaction time was calculated as the difference between stimuli presentation and the down keystroke. Both hit and FA reaction times were evaluated for incongruent flanker (hit), congruent flanker (FA), and n-back stimuli and then averaged over all individuals at each n-back level.

### Neurophysiological data

Both time-frequency analysis and ERP analysis were performed. These measures were evaluated only for the incongruent and congruent flanker trials with correct responses and averaged for each n-back level.

#### Event related potential (ERP) analysis

The positive potential occurring between 250 and 500 ms following stimulus presentation (P300) has been associated with allocation of attentional resources (Watter et al., 2001). Mean P300 amplitude was determined for both congruent and incongruent stimuli within this time window. The modulation of P300 amplitude have been observed over the parietal electrodes, thus Pz was used for this analysis (Watter et al., 2001; Polich, 2007). Mean P300 amplitude was determined for incongruent flanker trials at each n-back level.

#### Spectral analysis

A spectral analysis was performed over the 2–32 Hz frequency range for each participant and task condition. The frequency band power was calculated for the time window from 0 to 1,250 ms post-stimulus presentation, which has been observed to have maximal oscillatory effects for both updating and inhibiting executive functions (Scharinger et al., 2015). The effects of working memory load have been observed in the alpha band over the parietal electrodes, therefore the mean frequency band power for the alpha frequency was calculated at the Pz electrode for each participant and task condition (Gevins et al., 1997).

### Effective connectivity analysis

Following ICA and artifact rejection procedures, described above, all retained ICs were localized using DIPFIT. The Source Information Flow Toolbox (SIFT) for EEGLAB was used to evaluate effective connectivity, the causal flow of information between brain sources (Delorme et al., 2011). A multivariate autoregressive model (MVAR) was fit to the ensemble-normalized ICs using the Vieira-Morf algorithm with a 350 ms window length, 30 step size, and 39 model order. Model order was optimized from 1 to 40, such that the Hannan-Quinn criterion for each participant was minimized. Then the optimized model order values were averaged across all participants.

To validate the MVAR model, the whiteness of the residuals, model stability, and percent consistency were determined for each trial. The auto-correlation function (ACF) and the Li-McLeod (LMP) Portmanteau tests were used as whiteness test criteria. The LMP test was used due to its improved small-sample properties and lack of variance inflation compared to other available Portmanteau tests (Mullen, 2010). In addition to meeting the ACF and LMP criterion, the model stability was less than zero and percent consistency was above 85% for each trial, indicating a validated model.

Following model fitting and validation, SIFT was used to evaluate connectivity. The direct Directed Transfer Function (dDTF), a measure of frequency-domain conditional Granger causality, was estimated from the fitted model coefficients. The Directed Transfer Function (DTF) allows for analysis of short epochs of EEG activity to analyze information flow between different brain structures, while making it possible to determine spectral content of the signal (Kamiński et al., 2001). However, DTF is limited by its ability to differentiate between direct and indirect connections. By combining DTF and partial coherence measures, dDTF quantifies conditional, directionally-specific information transfer between sources over the trial time period at each frequency (Korzeniewska et al., 2003). In this study, dDTF was determined over the 2–32 Hz frequency range. A percentile threshold of 97.5% was used for each frequency to visualize relevant directional connections between brain sources.

### Procedure

All experiments were conducted in a quiet, dimly lit room where each participant was seated in a comfortable chair ~60 cm from a 17″ computer monitor. All participants were instructed to remain relaxed and avoid gross body movement during the experiment.

**Figure 2:**
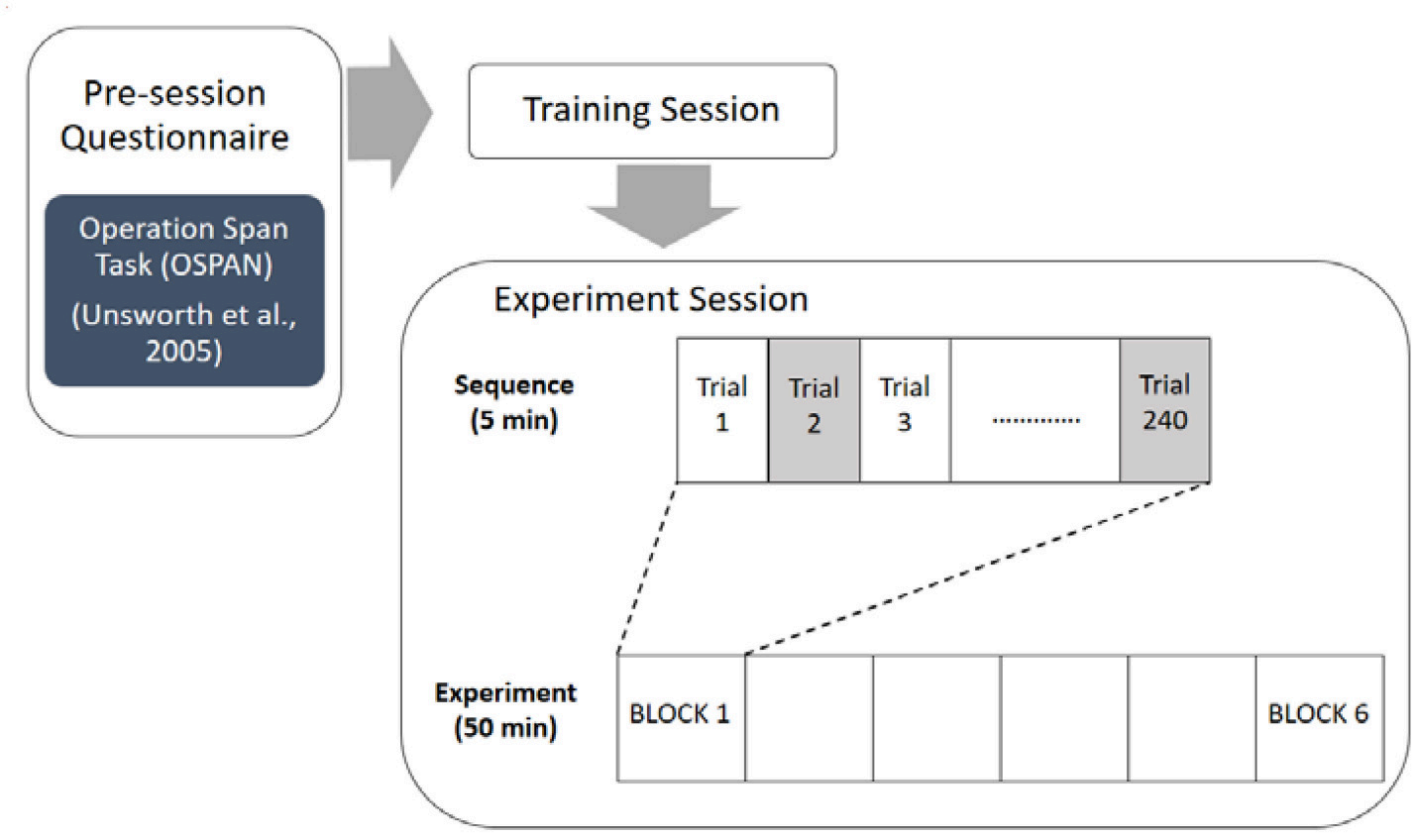
Experimental procedure.

After informed consent was given, participants were instructed to completed the automated version of the Operation Span task to assess participants' overall working memory capacity (Unsworth et al., 2005). Following the pre-experimentation survey session, participants proceeded through a training session in order to become familiarized with the experiment task (combined n-back and flanker task). Training was repeated until participants reached a criterion of no more than 36 errors out of the 60 trials (i.e., at least 60% correct responses) before they could move on to the main experiment. No tested participants failed to reach this criterion and had to repeat additional training trials. Afterwards participants performed the main experimental tasks. The experiment used a 3 × 2 repeated within-subjects factorial design featuring six conditions. Each participant completed six blocks, each block containing 240 alternating n-back and flanker trials (120 trials of each task). The overall flow of the experimental procedure is depicted in Figure 2. A 5 min break was given between each block. Block order was constant for each participant.

### Statistical analysis

Statistical analysis was performed in JMP (JMP, Version 12. SAS Institute Inc.) for behavioral and neurophysiological data. For all analyses, the level of significance was set to α = 0.05. In addition, effect size (η_*p*_^2^) and Macuchly's test of sphericity (ε) were reported. Every subject was exposed to an experiment with 2 (congruent, incongruent) × 3 (n-back level) mixed factor design. However, we decided to focus only on the data under the incongruent condition for relevance of neural connectivity study. Several studies (Nigbur et al., 2011; Scharinger et al., 2015) observed that the level of demand on inhibitory control (i.e., incongruent relative to congruent flanker trials) resulted in increased RTs, theta frequency band power, and pupil dilation as well as in decreased alpha frequency band power and accuracy. These studies have already compared congruent and incongruent trials. On the other hand, Klawohn et al. (2016) utilizes dual task (n-back + flanker) but only analyzes the main effect of the n-back level rather than comparing the flanker conditions (between congruent and incongruent). According to that, our study only focuses on incongruent trials for the relevance of neural connectivity analysis. For accuracy data, separate repeated measures analyses of covariance (ANCOVAs) were performed under incongruent flanker trials with factors n-back level (*n* = 0, 1, 2), and OSPAN score treated as a continuous covariate variable. (Conway et al., 2005) For reaction time data, ANCOVAs were performed only for incongruent Flanker trials with factors n-back level and OSPAN score. ANCOVAs also were performed for P300 amplitude and upper alpha band power with factors n-back level (*n* = 0, 1, 2) only for incongruent flanker condition and modulating factor of OSPAN score.

## Results

### Behavioral data

#### Accuracy (%)

During n-back test, for hit rate, we found significant main effects of n-back level [*F*_(2, 113)_ = 42.2776, *p* < 0.0001, ε = 0.808, η_*p*_^2^ = 0.631] and significant modulating effect of OSPAN [*F*_(1, 113)_ = 11.1574, *p* = 0.0011]. Table 2 shows mean value and standard deviation of hit and false alarm (FA) rates during n-back and flanker tasks. With the increase of n-back level (0-back: 98.4%, 1-back: 93.7%, 2-back: 79.6%; all n-back level Hit rate were significantly different), hit rate decreased during n-back test. Tukey's HSD (honest significant difference) showed that 2-back was significantly different with other two n-back levels (0-back and 1-back) for the hit rate of n-back test.

**Table 2:** Mean value and standard deviation of hit and false alarm (FA) rates.

	**N-back**		**Flanker**	
	**Hit rate (%)**	**False alarm rate (%)**	**Hit rate (%)**	**False alarm rate (%)**
0-back	0.984(0.03)	0.005(0.01)	0.998(0.01)	0.028(0.04)
1-back	0.937(0.08)	0.017(0.02)	0.997(0.01)	0.033(0.03)
2-back	0.796(0.14)	0.069(0.06)	0.989(0.02)	0.039(0.06)

As previously mentioned, the experimental design of current study was 2 (congruent, incongruent) x 3 (n-back level) mixed factor design. However, we only analyzed data under the incongruent condition for the flanker task. For the false alarm (FA) during n-back test, the main effect n-back level, showed significant effect [*F*_(2, 113)_ = 32.0015, *p* < 0.0001, ε = 0.555, η_*p*_^2^ = 0.408], but no significant modulating effect of OSPAN[*F*_(2, 113)_ = 1.442, *p* = 0.2327] was found. FA increased with the increase of n-back level (0-back: 0.5%, 1-back: 1.7%, 2-back: 6.9%). Tukey's HSD test showed that 2-back was significantly different with other two n-back levels (0-back and 1-back).

For the hit rate during flanker test, the main effect, n-back level [*F*_(2, 113)_ = 6.5119, *p* = 0.002, ε = 0.821, η_*p*_^2^ = 0.167] showed significance and marginally significant modulating effect of OSPAN was found[*F*_(2, 113)_ = 4.3889, *p* = 0.0385]. With the increase of n-back level, hit rate of flanker test decreased but the lesser degree compared with n-back hit rate (0-back: 99.8%, 1-back: 99.7%, 2-back: 98.9%). For the FA of flanker test, only OSPAN was found marginally significant modulating effect [*F*_(1, 113)_ = 7.3598, *p* = 0.0077].

#### Reaction time (RT, s)

Table 3 shows mean value and standard deviation of reaction times (RTs) on correct and incorrect responses during n-back and flanker tasks. RTs increased with increasing n-back levels.

**Table 3:** Mean value and standard deviation of reaction times (RTs) on correct and incorrect responses.

	**N-back**		**Flanker**	
	**RT correct (s)**	**RT incorrect (s)**	**RT correct (s)**	**RT incorrect (s)**
0-back	0.510(0.04)	0.522(0.24)	0.478(0.03)	0.389(0.07)
1-back	0.515(0.07)	0.568(0.11)	0.480(0.04)	0.405(0.07)
2-back	0.587(0.10)	0.636(0.14)	0.505(0.06)	0.371(0.08)

RTs of hit increased with the increase of n-back level (0-back: 510 ms, 1-back: 515 ms, 2-back: 587 ms; all n-back level RTs were significantly different). In addition, RTs of false alarm also increased with the increase of n-back level. (0-back: 522 ms, 1-back: 568 ms, 2-back: 636 ms) The main effect n-back level showed significant effect on RT of hit for n-back test [*F*_(2, 113)_ = 13.498, *p* < 0.0001, ε = 0.806, η_*p*_^2^ = 0.276], but non-significant effects were modulated by an individual working memory capacity, which is OSPAN score, [*F*_(1, 113)_ = 0.0481, *p* = 0.827]. The *post-hoc* analysis, Tukey's honest significant difference (HSD) test, showed that 2-back was significantly different with other two back levels (0-back and 1-back) for the RT of hit in n-back test. The n-back level effect showed significant effect on the RT of false alarm [*F*_(2, 113)_ = 1.636, *p* = 0.049, η_*p*_^2^ = 0.427] but no modulating effect. The Tukey HSD showed that all three levels of n-back were significantly different with each other for the RT of false alarm in n-back test.

During the flanker test, RTs of the hit increased with the increase of n-back level (0-back: 478 ms, 1-back: 480 ms, 2-back: 505 ms) and those outcome were significantly affected by main effects, n-back level [*F*_(2, 113)_ = 4.4740, *p* = 0.0136, ε = 0.653, η_*p*_^2^ = 0.210] and showed significant modulating effects of OSPAN [*F*_(1, 113)_ = 11.7384, *p* = 0.0009]. The *post-hoc* analysis, Tukey HSD test showed that 2-back was significantly different with other two back levels (0-back and 1-back) for the RT of hit during flanker test.

#### Correlation analysis

According to the Table 4, N-back level and hit rate have shown strongly negative correlation (*r* = −0.686, *p* < 0.005). False alarm rate during n-back task and n-back difficulty level presented positive correlation (*r* = 0.728, *p* < 0.005). Lastly, reaction time for n-back (*r* = 0.368, *p* < 0.01) also was significantly correlated with n-back level. In terms of OSPAN, n-back hit rate(*r* = 0.373, *p* < 0.01), flanker reaction time (*r* = −0.372, *p* < 0.01) and P300 amplitude (*r* = −0.387, *p* < 0.01) have shown significant correlation with OSPAN score.

**Table 4:** Correlation between n-back difficulty level, OSPAN score, behavioral data and neurological data (Pearson's correlation coefficient).

**Variables**	**N-back**	**OSPAN**	**Behavioral**						**Neurological**	
			**n-back Hit**	**n-back FA**	**Flanker hit**	**Flanker FA**	**n-back RT**	**Flanker RT**	**P300**	**UA**
N-back	1	0	−0.686^***^	0.728^***^	−0.359	0.018	0.368^*^	0.200	−0.324	−0.062
OSPAN		1	0.373^*^	0.106	0.196	0.251	0.032	−0.372^*^	−0.387^*^	0.083
n-back hit			1	0.324	0.324	−0.005	−0.287	−0.286	0.239	−0.034
n-back FA				1	−0.396^*^	0.094	0.154	0.203	−0.252	−0.059
Flanker hit					1	0.165	−0.217	−0.434^*^	0.013	−0.063
Flanker FA						1	−0.254	−0.529^*^	0.081	−0.137
n-back RT							1	0.464^*^	−0.037	−0.012
Flanker RT								1	0.157	0.186
P300									1	0.348
UA										1

Correlation is significant at the level

*** p < 0.005, ^**^p < 0.05,

* *p < 0.01 (2-tailed). Pearson's correlation was used, and sample size is (n = 19)*.

### Neurophysiological data

**Figure 3:**
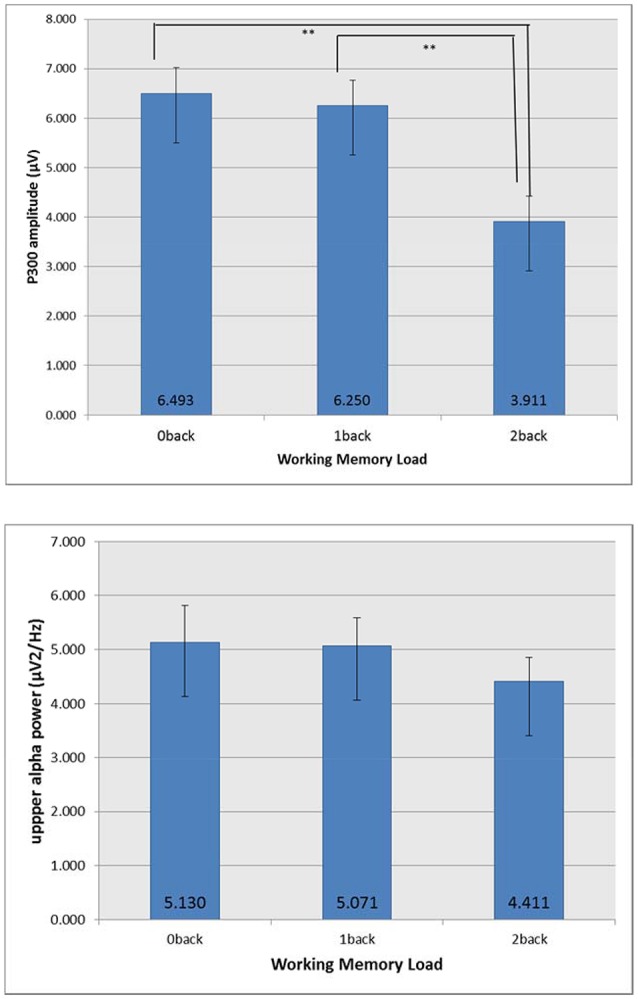
Mean P300 amplitude and upper alpha power at Pz. The error bar indicates the 98% confidence interval of one standard error. **p < 0.05.

For P300 amplitude (μV), with the increase of n back level, P300 amplitude decreased (0-back: 6.493, 1-back: 6.250, 2-back: 3.911). N-back level showed significant effect [*F*_(2, 95)_ = 154.6, *p* = 0.0009, ε = 0.938, η_*p*_^2^ = 0.394] but no modulating effect of OSPAN was found. Tukey's HSD test showed that 2-back was significantly different with other two n back levels (0-back and 1-back) for P300 amplitude. For the upper alpha power (μV^2^/Hz) (0-back: 5.130, 1-back: 5.071, 2-back: 4.411), no significant effect was found. Figure 3 shows mean P300 amplitude and upper alpha power at Pz. With the increase of n back level, P300 amplitude decreased. Tukey's HSD test showed that 2-back was significantly different with other two n back levels (0-back and 1-back) for P300 amplitude.

**Figure 4:**
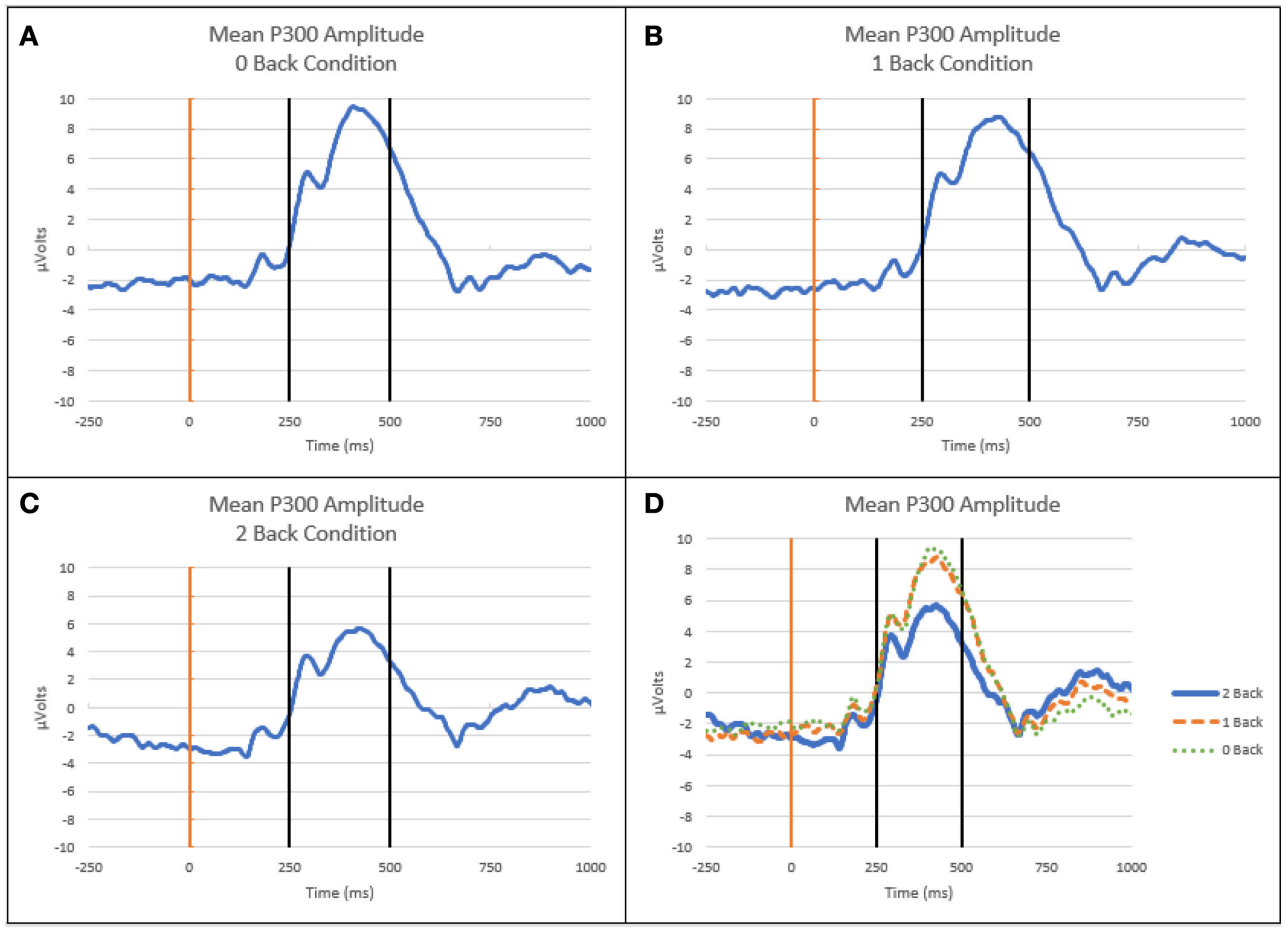
ERP waveforms at Pz electrode. **(A)** Mean ERP curve for 0-back level. **(B)** Mean ERP curve for 1-back level. **(C)** Mean ERP curve for 2-back level. **(D)** Mean ERP curves at all workload levels. Light vertical line is onset of Flanker stimulus, dark vertical lines indicate time window for mean P300 amplitude calculation (250–500 ms post-stimulus onset).

Mean P300 amplitude, as measured 250 to 500 ms post-stimulus on-set, over the parietal region decreased as cognitive workload (n-back level) increased. Figure 4 depicts the ensemble average EEG waveform for the Pz electrode on Flanker trials across all subjects at each workload level.

### Effective connectivity data

The data is common average referenced and zero phase high pass filtered at 0.1 Hz. The datasets were segregated into correct responses, time-locked to the button press, and separated into maximally independent components using Infomax ICA (Independent component analysis; Bell and Sejnowski, 1995). These sources were localized using a single or dual-symmetric equivalent-current dipole model using a four-shell spherical head model co-registered to the subjects' electrode locations by warping the electrode locations to the model head sphere using tools from the EEGLAB DIPFIT plug-in. Table 5 lists the cortical regions associated with Brodmann's areas (BAs) localized during 0-, 1-, and 2-back tasks.

**Table 5:** The cortical regions associated with Brodmann's area (BA) localized during 0-, 1-, and 2-back tasks.

	**0-back**	**1-back**	**2-back**
Brodmann's Area	• Prefrontal cortex (BA 10)	• DLPFC (BA 9)	• Parietal cortex (BA 5)
	• V1 (BA 17)	• Prefrontal cortex (BA 10)	• Prefrontal cortex (BA 10)
	• V2 (BA 18)	• V1 (BA 17)	• V1 (BA 17)
	• Temporal lobe (BA 21)	• V2 (BA 18)	• V2 (BA 18)
		• Temporal lobe (BA 21)	
	• Anterior cingulate cortex (BA 24)	• Parietal cortex (BA 39)	• Temporal lobe (BA 21)
	• Parietal cortex (BA 39)	• Thalamus (BA 50)	• Thalamus (BA 50)

For 0-back level, ICA revealed 7 independent components. Their dipoles were source localized to following regions: right middle temporal gyrus (BA 21), right primary visual cortex (V1) (BA 17), right ventral anterior cingulate (BA 24), left anterior prefrontal cortex (BA 10), right and left angular gyrus (BA 39) and left secondary visual cortex (V2) (BA 18). Under 1-back level, ICA revealed 8 independent components. Their dipoles were source localized to following regions: left secondary visual cortex (BA 18), right middle temporal gyrus (BA 21), right thalamus (BA 50), right primary visual cortex (BA 17), right anterior prefrontal cortex (BA 10), right and left angular gyrus (BA 39) and dorsolateral prefrontal cortex (DLPFC) (BA 9). For 2-back level, ICA revealed 6 independent components. Their dipoles were source localized to following regions: left secondary visual cortex (BA 18), right middle temporal gyrus (BA 21), right primary visual cortex (BA 17), right anterior prefrontal cortex (BA 10), right thalamus (BA 50), and right parietal cortex (BA 5).

#### Time-frequency distribution

**Figure 5:**
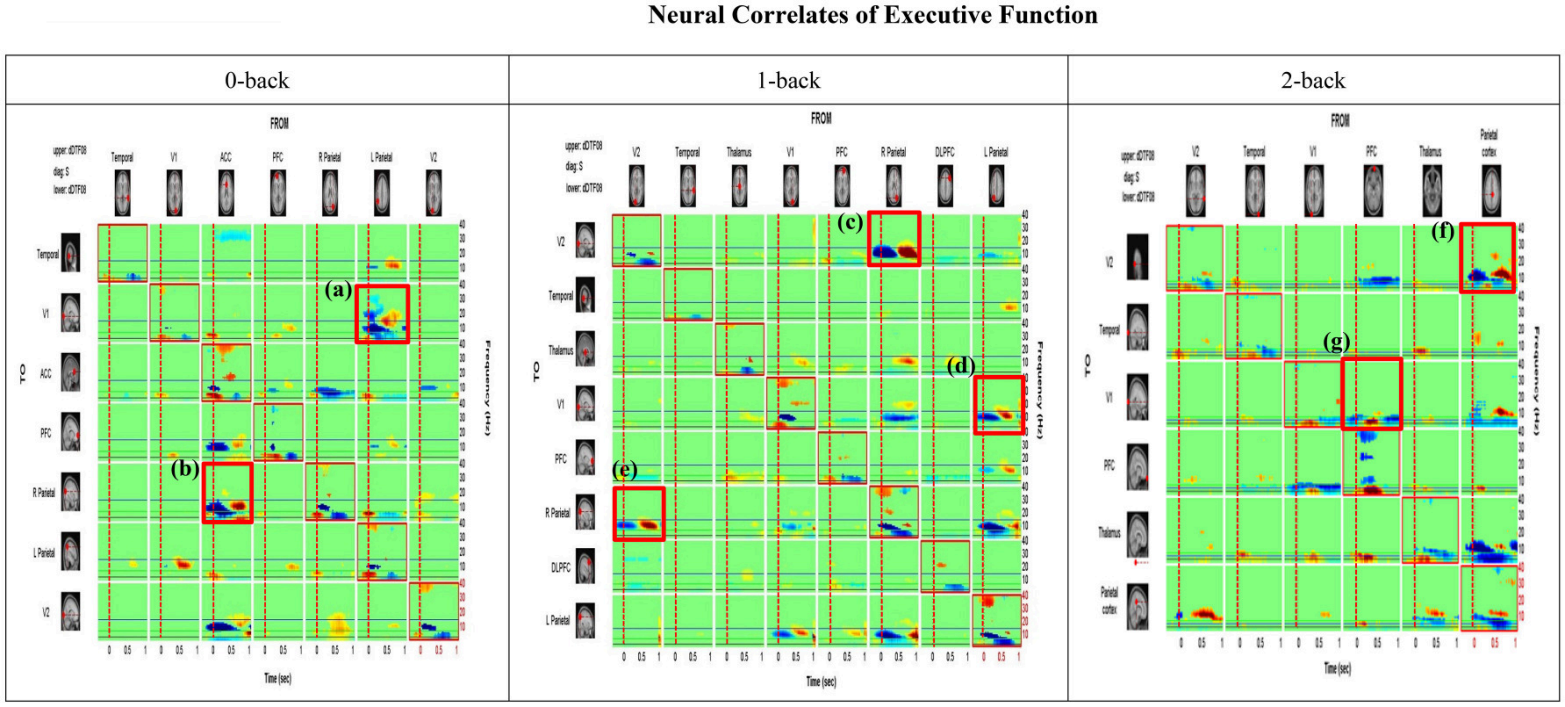
Time-Frequency Grid. Each cell of the matrix shows the time-frequency distribution of information transfer between a respective pair of ICs (i.e., columns represent the source or FROM and rows represent destination or TO), with the highest information transfer indicated by warm colors. The frequency is on the y-axis and time on the x-axis on each cell of the matrix. The upper and lower triangles of the grid (i.e., above and below the red-bordered diagonal cells, respectively) represent the dDTF (conditional GC) between each pair of sources (e.g., Visual cortex 2 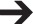
Visual cortex 1; Prefrontal cortex
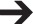
V2). Under the 0 back condition, the area (a) boxed in red [row 2, col 6] shows information flows at different times and frequencies from the source column 6 (Parietal) to the source row 2 (V1). The area (b) [row 5, col3] exhibits casual flow from the source column 3 (ACC) to the source row 5 (Parietal). On the other hand, under the 1 back condition, two boxed area (c) and (e) show bi-directional causal flows between V2 and right parietal cortex. The boxed area (d) indicates flows from the source column 8 (Left Parietal) to the source row 4 (V1). Lastly, under the 2 back condition, the boxed area (f) shows causal flows from the source column 6 (Parietal cortex) to the source row 1 (V2). The boxed area (g) presents information flows from the source column 4 (PFC) to the source row 3 (V1). The anatomical dipole locations for each source are rendered on the margins.

Mean causal information transfer (averaged across all participants) from each IC (column) to all other localized ICs (rows) as measured by the dDTF, is shown in Figure 5. Each cell of the matrix shows the time-frequency distribution (1–40 Hz) of information transfer between a respective pair of ICs, with highest information transfer indicated by warm colors.

Under the 0 back condition, the boxed area (a) highlighted in red [row 2, col 6] shows information flow at different times and frequencies from the source column 6 (Parietal) to the source row 2(V1). The boxed area (b) [row 5, col3] shows casual flow from the source column 3 (ACC) to the source row 5 (Parietal). On the other hand, under 1 back condition, two boxed area (c) and (e) show bi-directional causal flow between V2 and right parietal cortex. The boxed area (d) indicates flow form the source column 8 (Left Parietal) to the source row 4 (V1). Lastly, under the 2 back condition, the boxed area (f) shows causal flow from the source column 6 (Parietal cortex) to the source row 1 (V2). The boxed area (g) shows information flow from the source column 4 (PFC) to the source row 3 (V1). Dotted vertical lines indicate events of interest (the button-press) and horizontal blue lines denote 15 Hz frequencies. On the diagonal we have plotted the event-related spectral perturbation (ERSP).

The most notable feature of the analysis is that the PFC and parietal cortex are the strongest drivers of activity between other ICs. The PFC and parietal cortex are most strongly coupled to each other, but also demonstrate influence on the visual cortex and left and right parietal cortices. The greatest information flow was seen in the lower frequencies (3–15 Hz), which includes discrete theta and alpha bands with the highest information transfer observed in the theta band (3–7 Hz). Weaker reciprocal connectivity is apparent from temporal to occipital cortices.

#### Effective connectivity analysis

**Figure 6:**
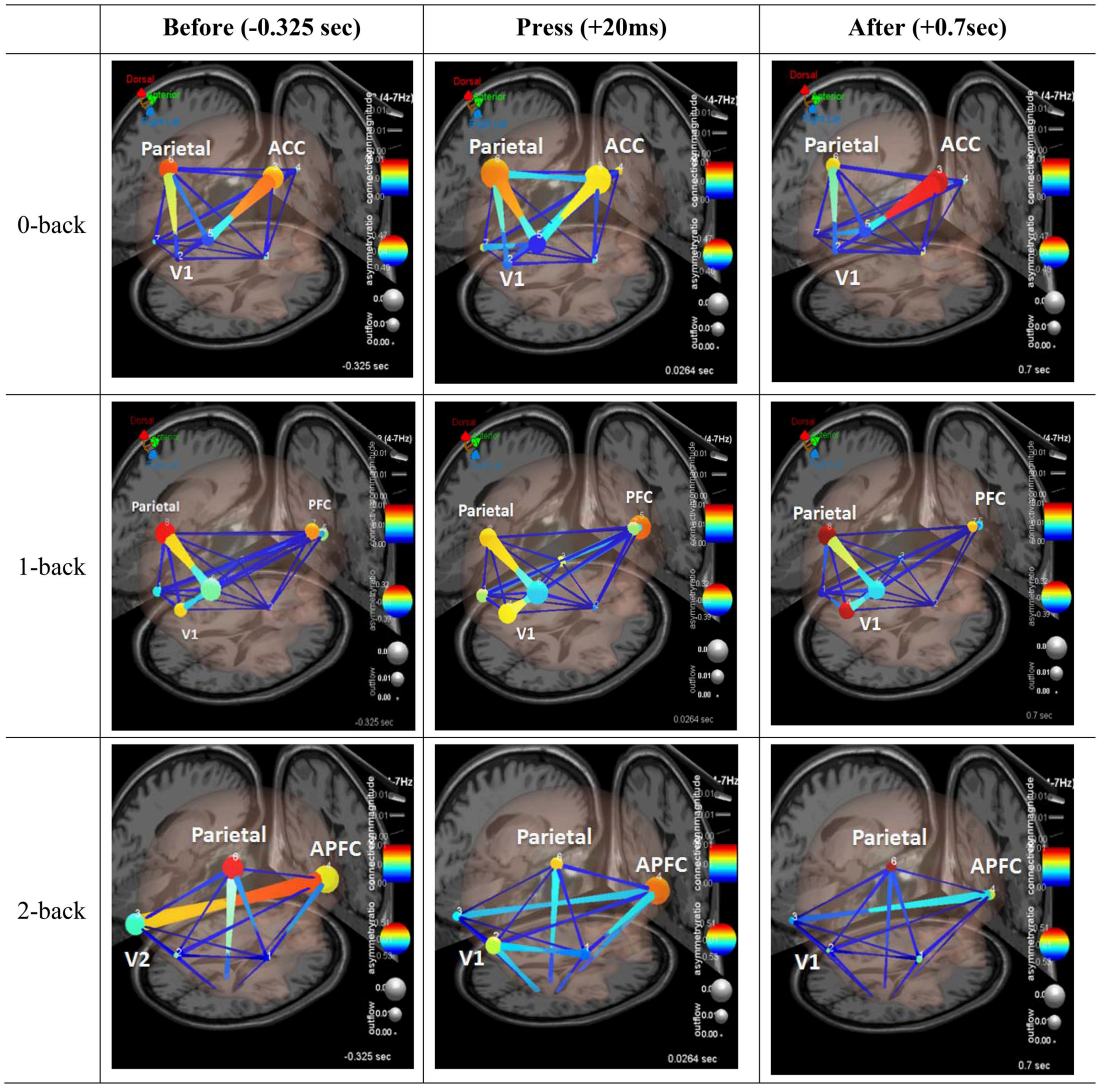
Effective connectivity analysis. Three frames of a causal brain maps show transient theta information flow during correct commission: The frames correspond to −325 ms (left), 20 ms (center), and 70 ms (right) relative to the button press (0 ms). The color of the edges represents connectivity strength (i.e., amount of information flow along that edge.). Red = high connectivity, Green = low connectivity. The size of edges of the graph represents connectivity magnitude (absolute value of connectivity strength). The color of node represents the asymmetry ratio of connectivity for that source. Red = causal source, blue = causal sink, green = balanced flow. The size of a node represents the amount of information outflow from the source.

We analyzed information flow between several of these anatomically localized sources of brain activity during trials with correct responses on incongruent flanker. Figure 6 shows three frames of a causal BrainMovie3D showing transient theta information flow during correct response. The frames correspond to −325 ms (left), 20 ms (center), and 70 ms (right) relative to the button press (0 ms). It is interesting to note that the theta rhythm (reflecting synchronized neural activity around the 4–7 Hz range) is associated with memory and cognitive function (Gevins et al., 1997; Tesche and Karhu, 2000) and has been related to decreased metabolism (Schacter et al., 1997).

Figure 6 shows the changes in causality over the time course of correct response events. For the first row, under the 0-back condition, parietal cortex and anterior cingulate cortex node color are red and orange. It indicated that those two components are the causal source for whole network. The edge size and color between visual cortex and parietal cortex coupling, and parietal cortex to prefrontal cortex coupling have red hue and thicker than the others. After button press, there is greater activation of the ACC and connectivity of the ACC and parietal cortex, as indicated by the deeper red color of the ACC node. Secondly, the second row, under the 1-back condition, it shows similar network pattern to 0-back condition. Before the button press, the causality flow was observed between parietal cortex and visual cortex. After button press, the coupling between prefrontal cortex and parietal cortex was activated. Lastly, under highest working memory load (2-back), different pattern from the previous condition was observed. From the −352 ms the anterior prefrontal cortex was activated as a causal source. As seen in Figure 6, there appeared to be enhanced coupling involving parietal cortex and prefrontal cortex across all time points. Moving to the time just following the button press event (center frame) we observed load-related specific patterns which have some is some bidirectional flow, but the flux is largely outward from parietal cortex, as indicated by the red hue of the node (indicating large positive asymmetry ratio).

## Discussion

The goal of this research was to better understand how two executive function processes (updating and inhibition) interact with each other. This study investigated this EF interaction at multiple levels of analyses (behavioral, neurophysiological, and effective connectivity).

### Effect of working memory load

As expected, increasing WM load on the updating task decreased the n-back task hit rate. This increase in WM load also decreased the hit rate for the flanker task, but to a lesser degree (ref. Table 2). The direction of these effects were significantly modulated by OSPAN score. For the neurophysiological metrics, updating level significantly affected P300 mean amplitude, however, this correlation is not modulated by OSPAN score. These observed effects in behavioral and electrophysiological measures for updating load concur with previous studies that utilized the n-back task, confirming that the used indicators are appropriate measures of WM updating load (Scharinger et al., 2015). In the correlation analysis, n-back level has significant correlation with behavioral data, such as n-back hit rate and reaction time and flanker task false alarm rate. OSPAN score also showed significant correlation with n-back hit rate, reaction time for the flanker task and mean P300 amplitude.

### Validation for cognitive control network (CCN)

We hypothesized that source localization would identify neural sources within the CCN in the frontal and parietal areas. Additionally, it was expected that the neural sources involved in correct Flanker responses with high updating load conditions (2-back) would exhibit enhanced activation of the CCN as compared to the connectivity observed in low updating load conditions (0-back and 1-back). Our finding conformed to the model proposed by Baddeley (1996b), which was proven from numerous imaging studies on effective connectivity of healthy subjects that have identified cortical areas particularly associated with the working memory network (Cabeza et al., 1997; Della-Maggiore et al., 2000; Glabus et al., 2003). Executive control functions are reported to be sub-served by the anterior cingulate cortex and the prefrontal cortex (BA9 and 10), whereas preprocessing and maintenance of information mainly involve activation of the parietal association cortex (BA 39) (Chee and Choo, 2004). The current study showed the change of the connectivity strength inside the executive subsystem of the working memory networks. There is growing evidence that attentional processes may recruit structures in superior parietal and anterior cingulate areas among others (Courtney et al., 1996). Taken together, these hypotheses account for the majority of cognitive processes involved in the n-back and flanker task.

### Effective connectivity along with updating demand

Granger causality methods were utilized to study dynamic interactions between prefrontal and parietal cortices. Granger causality between these two regions can be defined as the extent to which data from one region at one point in time improve the prediction of another region's data at a later point in time (Goebel et al., 2003). Using GC analysis of EEG time series data, the current study demonstrated that manipulation of cognitive demand in dual tasks influenced the updating and inhibition process. The main result of this study was in line with our original hypothesis that experimental manipulation of WM load in the n-back paradigm would cause changes in the integrated function of the large-scale neurocognitive network.

We used Granger Causality analysis to model a set of directional or effective connections between frontal and parietal cortical areas, and found evidence for load-related modulation of effective connectivity patterns. Referring to Figure 6, the present study compared connectivity patterns for correct, incongruent Flanker responses, before, during, and immediately following response. Under the 0-back level, a causal flow exhibited a V-shape pattern, with connectivity between visual, parietal, and prefrontal cortices (visual cortex → parietal cortex → prefrontal cortex). Under the 1-back level, patterns showed an angle bracket shape (“>”) (visual cortex → parietal cortex → prefrontal cortex). However, under the 2-back level, patterns displayed a substantially different shape, with the causal flow exhibiting a cross shape (“+”). Even before participants pushed the button, the causal flow activated visual cortex → prefrontal cortex and parietal cortex → thalamus. The changes in causal flow patterns depending on memory load supported the over additive hypothesis; high working memory load can induce an efficient brain network for simultaneous activation of visual, parietal, prefrontal cortex.

This study demonstrated that an interaction between inhibition and updating functions became hyper-synchronized during the experiment. As one set of trials ended and moved into a post-phase, hyper-coupling subsided and returned to normal. Constant hyper-coupling among the prefrontal cortex, parietal cortex and visual cortex seemed to appear during this transition. Hyper-coupling seemed to be associated with an increase in causality from posterior to anterior regions. There was also a pervasive pattern of low connectivity at and between regions including temporal lobe. It was clear that there were multiple, complex connectivity relationships involved in these processes that need to be understood better.

### Major causal flow during dual task

Rypma and Prabhakaran (2009) argued that the neural mechanism has a cost-efficient component which causes prefrontal cortex to exert more influence over other brain regions. Although, their founding was subject to individual capacity, intrapersonal analysis suggested that the extent of direct processing links between neural nodes determined the efficiency of working memory load. The benefit of the direct processing links stemmed from a surplus of resources that maximize available capacity permitting better performance in upper alpha power.

As shown in Figure 6, PFC along with parietal cortex served as a main hub for the cognitive network. This finding concurs with that of previous studies that have shown that PFC contains WM-specific sensitivity lesions (Quintana and Fuster, 1993; Rypma and Prabhakaran, 2009). The current study showed that PFC under high WM load exhibited stronger causality than one under low WM load. The relationship between PFC and WM load suggests the importance of trainings needed for those working in a dangerous working environment that often requires complex multitasking under stress. This is supported by Olesen et al. (2004) that trainings of working memory increased activity levels in prefrontal and parietal regions.

## Limitation and future research

The results of the current study indicated that if EFs are specifically loaded, rather than a reduction of attentional processes, an enhanced activation of attention control might occur, thus leading to a different CCN patterns under high WM updating load. Moreover, under high WM load the CCN exhibited a differential activation pattern as compared with the low WM load, which may be indicative of a more efficient causal flow between the two executive function processes. Certainly, the current study can only serve as a first step in studying the interplay between different EF components when manipulated within one single task, therefore, there remain open questions to be addressed in more detail in future studies. First, the sample size of this study is relatively small. While a power analysis supports the use of 19 subject to analyze and draw conclusions from the covariance of performance and neurophysiological metrics, the findings of this study would be more strongly supported if future studies using a similar paradigm utilize a larger sample size. Next, simple cognitive tasks, the flanker and n-back tasks, were utilized in this study to avoid the “impurity issue” and ensure direct investigation of updating and inhibition executive functions (Miyake et al., 2000). However, these tasks may lack ecological validity and the context usually associated with real-world tasks. To allow for more wide-spread transferability of the connectivity analysis, future research paradigms should aim to modify real-world stimuli or tasks. Additionally, while subject OSPAN score was found to be a main effect in both behavioral and neurophysiological metrics, the direction of the OSPAN effect was not explored. Future studies should aim to characterize the directional effect of high and low working memory capacity on performance neurophysiological responses during dual EF tasks. Furthermore, the impact of WM capacity could not be investigated in the GCA analysis due to the small sample size. This analysis may reveal differential connectivity patterns correlated with behavioral or neurophysiological metrics.

Activation of distinct brain regions to perform a specific task, as observed in this study, can be modeled as a network with each node being a single brain region and each edge representing interaction between brain regions. This study utilized the dDTF as a measure of Granger Causality to analyze the time-varying interaction between brain areas at different n-back levels. While GCA allows for a statistical analysis of the EEG time-series data to identify and characterize causal relationships, this analysis method lacks regard for the underlying structural connectivity of the brain (Seth et al., 2015). Other modeling methods, such as dynamic causal modeling, require a more detailed definition and analysis of the underlying neural structure and physical mechanisms underlying a specific response (Friston et al., 2013; Seth et al., 2015).

Furthermore, GCA provides a characterization of interactions between brain areas, with no analysis of the efficiency of this interaction. Brain networks can also be modeled as “small-worlds,” as they have clustered local connectivity with short path lengths between regions in the network (Latora and Marchiori, 2001; Achard and Bullmore, 2007). This small-world model of brain networks can support analysis of different means of information processing and of network efficiency. Analysis of these functional networks through MRI data, support the hypothesis of the small-world properties, including high performance at a low cost, termed efficiency. Brain network efficiency is impacted by both individual factors, such as age, pharmacological blockades of dopamine transmission (Latora and Marchiori, 2001) and diseases such as multiple sclerosis (He et al., 2009). While GCA is a powerful analysis method to investigate cause and effect relationships between brain regions during task performance, it is not without limitations. To expand our knowledge of the interplay between updating and inhibition, including the role of the capacity of attentional resources, different modeling techniques, and measures of network connectivity should be explored.

## Concluding remarks

This research explored how two executive function processes (updating and inhibition) interacted with each other. The study investigated interaction between the two executive function components through analyses of behavioral, neurophysiological, and effect connectivity metrics. Using Granger Causality analysis of EEG time series data, the study demonstrated that manipulation of cognitive demand in a dual executive functions task influenced the updating and inhibition process. Specifically, the experimental manipulation of working memory load in the n-back paradigm would increase the integrated function of a large-scale neurocognitive network, which contains prefrontal and parietal cortices. These results provide insights into the neural mechanisms underlying cognition, specifically the relationship between unitary executive functions that are building blocks of complex cognition. Future applications of these results include task design and working memory training for human operators performing complex, multi-tasking operations in stressful environments.

## Author contributions

All authors listed, have made substantial, direct and intellectual contribution to the work, and approved it for publication.

### Conflict of interest statement

The authors declare that the research was conducted in the absence of any commercial or financial relationships that could be construed as a potential conflict of interest. The reviewer XP and handling Editor declared their shared affiliation, and the handling Editor states that the process nevertheless met the standards of a fair and objective review.
